# Functional hemodynamic tests: a systematic review and a metanalysis on the reliability of the end-expiratory occlusion test and of the mini-fluid challenge in predicting fluid responsiveness

**DOI:** 10.1186/s13054-019-2545-z

**Published:** 2019-07-29

**Authors:** Antonio Messina, Antonio Dell’Anna, Marta Baggiani, Flavia Torrini, Gian Marco Maresca, Victoria Bennett, Laura Saderi, Giovanni Sotgiu, Massimo Antonelli, Maurizio Cecconi

**Affiliations:** 1 Department of Anesthesia and Intensive Care Medicine, Humanitas Clinical and Research Center – IRCCS, Via Alessandro Manzoni, 56, 20089 Rozzano, MI Italy; 2 Department of Anesthesiology and Intensive Care Medicine, Catholic University of the Sacred Heart, Fondazione “Policlinico Universitario A. Gemelli”, Rome, Italy; 3 Fondazione Policlinico Universitario A. Gemelli IRCCS, Rome, Italy; 4 Department of Anesthesiology and Intensive Care Medicine, A.O.U. Maggiore della Carità, Novara, Italy; 5 Department of Intensive Care Medicine, St George’s University Hospital NHS Foundation Trust, London, UK; 6 Clinical Epidemiology and Medical Statistics Unit, Department of Biomedical Sciences, University of Sassari, Research, Medical Education and Professional Development Unit, AOU Sassari, Sassari, Italy; 7 Department of Biomedical Sciences, Humanitas University, Pieve Emanuele, MI Italy

**Keywords:** Functional hemodynamic test, Fluid responsiveness, End-expiratory occlusion test, Mini-fluid challenge

## Abstract

**Background:**

Bedside functional hemodynamic assessment has gained in popularity in the last years to overcome the limitations of static or dynamic indexes in predicting fluid responsiveness. The aim of this systematic review and metanalysis of studies is to investigate the reliability of the functional hemodynamic tests (FHTs) used to assess fluid responsiveness in adult patients in the intensive care unit (ICU) and operating room (OR).

**Methods:**

MEDLINE, EMBASE, and Cochrane databases were screened for relevant articles using a FHT, with the exception of the passive leg raising. The QUADAS-2 scale was used to assess the risk of bias of the included studies. In-between study heterogeneity was assessed through the *I*^2^ indicator. Bias assessment graphs were plotted, and Egger’s regression analysis was used to evaluate the publication bias. The metanalysis determined the pooled area under the receiving operating characteristic (ROC) curve, sensitivity, specificity, and threshold for two FHTs: the end-expiratory occlusion test (EEOT) and the mini-fluid challenge (FC).

**Results:**

After text selection, 21 studies met the inclusion criteria, 7 performed in the OR, and 14 in the ICU between 2005 and 2018. The search included 805 patients and 870 FCs with a median (IQR) of 39 (25–50) patients and 41 (30–52) FCs per study. The median fluid responsiveness was 54% (45–59). Ten studies (47.6%) adopted a gray zone analysis of the ROC curve, and a median (IQR) of 20% (15–51) of the enrolled patients was included in the gray zone. The pooled area under the ROC curve for the end-expiratory occlusion test (EEOT) was 0.96 (95%CI 0.92–1.00). The pooled sensitivity and specificity were 0.86 (95%CI 0.74–0.94) and 0.91 (95%CI 0.85–0.95), respectively, with a best threshold of 5% (4.0–8.0%). The pooled area under the ROC curve for the mini-FC was 0.91 (95%CI 0.85–0.97). The pooled sensitivity and specificity were 0.82 (95%CI 0.76–0.88) and 0.83 (95%CI 0.77–0.89), respectively, with a best threshold of 5% (3.0–7.0%).

**Conclusions:**

The EEOT and the mini-FC reliably predict fluid responsiveness in the ICU and OR. Other FHTs have been tested insofar in heterogeneous clinical settings and, despite promising results, warrant further investigations.

**Electronic supplementary material:**

The online version of this article (10.1186/s13054-019-2545-z) contains supplementary material, which is available to authorized users.

## Introduction

Tailored fluid therapy has received increasing attention in the management of patients with acute circulatory failure in both the intensive care unit (ICU) and operating room (OR). The aim is to try and prevent both inadequate tissue perfusion and fluid overload [1]. Unnecessary fluid administration has been associated with increased morbidity, mortality, and hospital length of stay in both critically ill and surgical patients [2–10].

The only physiological reason to give a fluid challenge (FC) to a patient with acute circulatory failure is to increase the stroke volume (SV) ultimately leading to an increase in oxygen transport [11–13]. However, this is only achieved in approximately 50% of ICU and OR patients [14, 15]. The prediction of fluid responsiveness prior to FC administration is a topic of interest, which has been extensively investigated, but remains challenging [1, 13, 16–18]. Bedside clinical signs, systemic pressures, and static volumetric variables poorly predict fluid responsiveness [17]. Moreover, the values of the ventilator-induced dynamic changes in pulse pressure and stroke volume [pulse pressure variation (PPV) and stroke volume variation (SVV), respectively] are often unreliable in a significant number of ICU and OR patients [19–21].

To overcome these limitations, bedside functional hemodynamic assessment has gained in popularity [17, 18, 22]. A functional hemodynamic test (FHT) consists of a maneuver that affects cardiac function and/or heart-lung interactions, with a subsequent hemodynamic response, the extent of which varies between fluid responders and non-responders [17, 18, 22].

The FHT called passive leg raise (PLR) has been successfully used since 2009 to assess fluid responsiveness in ICU patients [23], as confirmed by three metanalyses [24–26]. Some conditions, however, including abdominal or intracranial hypertension and traumatic hip or lower limb fractures, limit the use of a PLR [27], and it is often unfeasible in the OR.

A number of different FHTs have been proposed as alternatives to the PLR, for use in both the ICU and more recently the OR. These tests can be subdivided into two groups. One subgroup of FHTs is based on the assessment of changes in systemic PPV and SVV or left ventricular SV in response to a predefined alteration in ventilatory settings. These tests rely on physiological heart-lung interactions, which can affect several cardiac properties. A change in respiratory dynamics alters venous return, leading to changes in right ventricular preload, afterload, and subsequently left ventricular function. [23, 28]. A second subgroup of tests aims at testing the increase in SV after the rapid administration of a small aliquot of a predefined FC [29, 30].

Since the reliability and the limits of PPV, SVV, and PLR in predicting fluid responsiveness have been already extensively investigated in different clinical settings [15, 24–26, 31], we conducted a systematic review of the literature and performed a metanalysis aimed at assessing the overall quality, external validation, consistency, and risk of bias of the other FHTs available in both the ICU and OR.

## Material and methods

### Study selection and inclusion criteria

We included articles published in the English language, in indexed scientific journals, from 1966 to June 2018. Reviews, case reports, and studies published in abstract form were not included. Only studies performed in adults were eligible for inclusion.

Only studies that compared the reliability of the FHT to a FC, as the gold standard for assessing fluid responsiveness, were included. The definition of a FHT was a standardized hemodynamic maneuver affecting cardiac function and/or heart-lung interactions and used to assess fluid responsiveness. The definition of a FC was a fixed quantity of fluid administered in a defined time to change a hemodynamic variable by a predetermined threshold. We included only the following hemodynamic variables as potential indicators of a positive FC: cardiac output (CO); SV; their indexed values (CI and SVI) or SV surrogates, i.e., aortic velocity-time integrals; and aortic blood flow, as assessed by either transthoracic or trans-esophageal echocardiography.

We excluded those studies in which FHTs were performed in patients with an open chest or with atrial fibrillation. We did not impose exclusion criteria regarding the modality or the absence of mechanical ventilation.

### Search strategy and data extraction

We independently searched the MEDLINE, EMBASE, and Cochrane Database of Systematic Reviews using the following search criteria: (fluid AND responsiveness) OR passive AND leg AND raising) OR end-expiratory AND occlusion AND test) OR pulse AND pressure AND variation) OR stroke AND volume AND variation) OR (dynamic AND indices OR indexes)) OR mini-fluid challenge) OR functional AND hemodynamic AND monitoring) OR (fluid AND challenge). Filters: Humans; English; Adult: 19+ years.

The references for all included papers, review articles, commentaries, and editorials on this topic were also reviewed to identify other studies of interest that were missed during the primary search. Two of the authors (FT and GM) independently performed the evaluation of titles and abstracts. The articles were then subdivided into three subgroups: “included” and “excluded” (if the two examiners agreed with the selection) or “uncertain” (in case of disagreement). In case of “uncertain” classification, a further examination was performed by an expert (AM) and a conclusive decision was made.

We used a standardized data form to extract the data from all included studies, recording (1) the characteristics of the investigated population, (2) the methods used to perform the FHT test and to assess its hemodynamic effect, (3) the modalities of FC administration and the definition of fluid responsiveness, and (4) the area under the receiver operating characteristic (ROC) curve (AUC) and all the statistical data obtained by the ROC curve analysis (i.e., sensitivity, specificity, Youden index, positive and negative predictive values, positive and negative likelihood ratios). For those studies in which more than one method of hemodynamic monitoring was used to estimate flow parameters, we reported only the data obtained by the technique considered to be the most reliable, according to the following scale: pulmonary artery catheter or calibrated technique > cardiac echocardiography performed by experts (both transthoracic or trans-esophageal) > uncalibrated technique or esophageal Doppler probes > bioimpedance or bioreactance.

### Assessment of risk of bias in the included studies

The QUADAS-2 scale was used to assess the risk of bias of the included studies [32]. Two expert authors (AM and MC) independently examined the studies using predefined criteria, which are reported in Additional file 1: Table S1.

For each criterion, the risk of bias was judged as high (3 points), unclear (2 points), or low (1 point). If the answers to all signaling questions for a domain were “yes,” then the risk of bias was judged as “low.” If any signaling question was answered “no,” the potential risk of bias was defined as indicated in Additional file 1: Table S1. The sum of these points was used to calculate the global risk of bias.

Studies were included in the highest risk of bias group if the sum of the points obtained by the risk of bias and applicability judgment assessment was higher than the median value for all the studies.

### Statistical analysis

Statistical analysis was conducted on the summary statistics described in the selected articles (e.g., means, medians, proportions), and therefore, the statistical unit of observation for all the selected variables was the single study and not the individual patients.

The descriptive statistics of individual studies used different statistical indicators for central tendency and variability, whereas absolute and relative frequencies were adopted for qualitative variables. Quantitative variables were summarized with means (standard deviation, SD) or medians (25th–75th interquartile range, IQR) according to their distribution.

For the selected studies, we planned to perform (1) a metanalysis in order to determine the pooled AUC and the pooled sensitivity and specificity of the FHT as a predictor of fluid responsiveness and (2) a metanalysis in order to determine the pooled correlation between the changes in the flow hemodynamic parameters after FHT and the changes after FC administration. The FC was the exposure variable, and clinical and hemodynamic characteristics were considered as the outcome variables. Fixed effect models were used. In-between study heterogeneity was assessed through the *I*^2^ indicator. Bias assessment graphs were plotted, and Egger’s regression analysis was used to evaluate the publication bias. Student’s *t* test or Mann-Whitney test for parametric or non-parametric distributions were respectively used to assess a difference in mean values between responders and non-responders.

Statistical analyses were conducted using GraphPad PRISM V6 (GraphPad Software Inc., San Diego, CA, USA) and STATA®13 (StataCorp, College Station, TX, USA). For all comparisons, we considered *p* values < 0.05 significant.

## Results

**Fig. 1:**
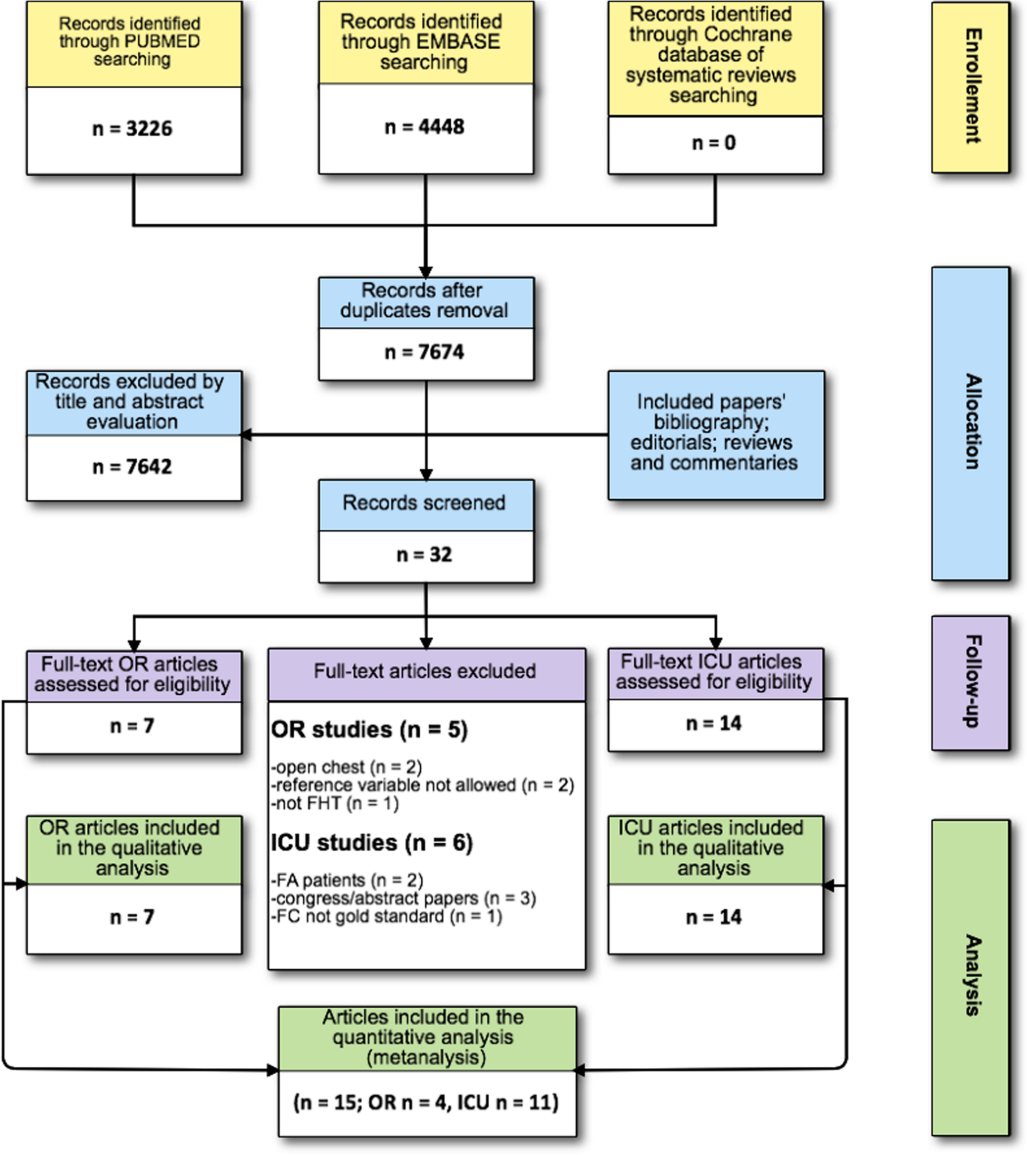
Flow of the studies. FC, fluid challenge; ICU, intensive care unit; FA, atrial fibrillation; OR, operating room; FHT functional hemodynamic test

The electronic search identified 7674 titles. After the first assessment by two authors, 32 full-text manuscripts were included in the secondary analysis and 21 met the inclusion criteria: 7 performed in OR and 14 in ICU between 2005 and 2018. The senior examiner evaluated 177 of the 7524 (2%) potentially relevant studies because of disagreement between the two authors. A detailed description of the selection process flow is provided in Fig. 1. We did not find any further relevant publications by reviewing the references of the selected studies, review articles, commentaries, or editorials regarding the use of FHTs.

According to the search criteria, we identified seven different types of FHTs (see Table 1):

1. An interruption of the mechanical ventilation for few seconds to determine an increase in right ventricle preload (the end-expiratory occlusion test EEOT)
2. A quick administration of an aliquot of 50–100 ml of fluid to increase the SV (the mini-FC test)
3. The use of a lung recruitment maneuver (LRM) of 25–30 cmH_2_0 to affect the hemodynamic response of the right ventricle
4. The assessment of the systolic arterial pressure decrease after the use of successive incremental pressure-controlled breaths [the respiratory systolic variation test (RSVT)]
5. The assessment of the arterial pressure response during a Valsalva maneuver
6. The assessment of the arterial pressure elevated during a brief increase of the positive end-expiratory pressure increase from 10 to 20 cmH_2_0
7. An increase of the tidal volume from 6 to 8 ml/kg for 1 min to enhance the baseline reliability of the dynamic indexes of fluid responsiveness

**Table 1:** Characteristics of the functional hemodynamic tests and of the fluid challenges

Studies		General characteristics					FC characteristics						
		Patients	FCs	Months	Ventilatory setting	FHT: description	Volume infused (ml)	Time (min)	Rate (ml/min)	Reference variable	Type of fluid	Hemodynamic monitoring	FR (%)
Operating room	Guinot et al. [33]	42	42	NS	CMV	EEOT: ventilation interrupted for 15 s	500	10	50	SV > 15%	Ringer	CardioQ	67
	Biais et al. [34]	41	41	6	CMV	EEOT: ventilation interrupted for 30 s	250	10	25	SVI ≥ 10%	Saline	ProAQT	51
	Biais et al. [35]	44	88	12	CMV	Mini-FC: 100 ml in 120 s	250	10	25	SVI ≥ 10%	Saline	ProAQT	32
	Guinot et al. [36]	73	73	6	SB	Mini-FC: 100 ml in 60 s	500	10	50	SV > 15%	Ringer	NICCOMO	37
	Preisman et al. [37]	18	70	NS	CMV	RSVT: decrease in SAP after successive incremental pressure-controlled breaths (10, 20, and 30 cmH_2_O)	250	5	50	SVI ≥ 15%	Poligeline 3.5%	PiCCO	46
	Biais et al. [38]	28	28	12	CMV	LRM: 30 cmH20/30 s	250	10	25	SVI ≥ 10%	Saline	ProAQT	57
	De Broca et al. [39]	60	60	9	CMV	LRM: 25 cmH20/25 s	500	10	50	SV ≥ 15%	Ringer	CardioQ	62
Intensive care unit	Wu et al. [40]	50	50	8	CMV	Mini-FC: 50 ml in 10 s	500	15	33	CO ≥ 15%	Crystalloids (undefined)	TTE	54
	Smorenberg et al. [41]	21	21	NS	CMV	Mini-FC: 100 ml in 120 s	500	20	25	CO > 10%	Hydroxyethyl starch 6%	Modelflow/PulseCO	62
	Muller et al. [29]	39	39	10	CMV	Mini-FC: 100 ml in 60 s	500	15	33.3	VTI ≥ 15%	6% HES	TTE	54
	Monge Garcia et al. [42]	30	30	6	SB	Airway pressure elevated up to 30 cmH20 for 10 s	500	30	16.6	SVI ≥ 15%	6% HES	Flow-Trac	37
	Perel et al. [28]	14	14	NS	CMV	RSVT: decrease in SAP after successive incremental pressure-controlled breaths (5,10, 15, and 20 cmH_2_O)	7/kg	30	NA	CI ≥ 15%	Plasma expander (undefined)	PAC	57
	Yonis et al. [43]	33	33	40	CMV	EEOT: ventilation interrupted for 15 s	500	15	33.3	CI ≥ 15%	Crystalloids (undefined)	PiCCO	45
	Xiao-ting et al. [44]	48	48	8	CMV	Mini-FC: 100 ml in 60 s	500	15	33.3	CI ≥ 10%	Saline	PiCCO	71
	Mallat et al. [45]	49	49	NS	CMV	Mini-FC: 100 ml in 60 s	500	15	33.3	CI ≥ 15%	4% albumin	PiCCO	45
	Georges et al. [46]	50	50	NS	ACV(V); no SE	EEOT: ventilation interrupted for 12 s	500	15	33.3	CO ≥ 15%	Saline	TTE	56
	Wilkman et al. [47]	20	20	NS	CMV	PEEP elevated from 10 to 20 cmH_2_O for 60–120 s	6/kg	30	NA	CO ≥ 15%	Succinilgelatine 4%	TEE	30
	Jozwiak et al. [48]	30	30	14	ACV(V); no SE	EEOT: ventilation interrupted for 15 s	500	10	50	CI > 15%	Saline	PiCCO	50
	Monnet et al. [23]	34	34	NS	ACV(V); SE	EEOT: ventilation interrupted for 15 s	500	10	50	CI > 15%	Saline	PiCCO	68
	Myatra et al. [49]	20	30	NS	ACV(V); no SE	*V*t raised up from 6 to 8 ml/kg for 1 min	7/kg	10	NA	CI > 15%	Saline	PiCCO	53
	Monnet et al. [50]	54	54	NS	ACV(V); no SE	EEOT: ventilation interrupted for 15 s	500	20	25	CI ≥ 15%	Saline	PiCCO	55

*FC* fluid challenge, *CMV* controlled mechanical ventilation, *ACV(V)* volume-assist controlled mechanical ventilation, *SB* spontaneously breathing patients, *SE* spontaneous efforts, *EEOT* end-expiratory occlusion test, *V*_*t*_ tidal volume, *MV* mechanical ventilation, *LRM* lung recruitment maneuver, *RSVT* respiratory systolic variation test, *SAP* systolic arterial pressure, *PEEP* positive end-expiratory pressure, *CO* cardiac output, *CI* cardiac index, *SV* stroke volume, *SVI* stroke volume index, *FR* fluid responsiveness, *HES 6%* hydroxyethyl starch 6%, *TEE* trans-esophageal echocardiography, *TEE* transthoracic echocardiography, *PAC* pulmonary artery catheter; *NA* not applicable; *CardioQ*, Deltex Medical Ltd., Chichester, UK; *PiCCO/ProAQT*, PULSION Medical Systems; *FloTrac*, Edwards Lifesciences, Irvine, CA, USA; *NICCOMO*, non-invasive continuous cardiac output, Imedex, France; *PulseCO*, LiDCOltg, Cambridge, UK; *Modelflow*, FMS, Amsterdam, the Netherlands

All the studies were monocentric and, overall, included 805 patients and 870 FCs with a median (IQR) of 39 (25–50) patients and 41 (30–52) FCs per study. The median (IQR) fluid responsiveness was 54% (45–60) and was not different between the OR and ICU studies [51% (37–62) vs. 54% (45–58), respectively; *p* = 0.81]. The hemodynamic values of responders and non-responders before FHT application in both the OR and ICU studies did not differ (see Additional file 1: Table S2). Ten studies (48%) adopted a gray zone analysis of the ROC curve, and a median (IQR) of 20% (15–51) of the enrolled patients was included in the gray zone.

Overall, the median (IQR) QUADAS-2 score of the included studies was 9 (8–11) and was not different between the OR and ICU [10 (8–11) vs. 9 (8–11), respectively; *p* = 0.67]. Three OR studies (43%) and six ICU studies (43%) were classified in the subgroup with the highest risk of bias (see Table 2).

**Table 2:** QUADAS-2 score assessment of the included studies

Studies		Patient selection					Index test					Reference standard					Flow and timing		Final score	Final risk
		Risk of bias		Applicability judgments		TOT	Risk of bias		Applicability judgments		TOT	Risk of bias		Applicability judgments		TOT				
Operating room	Guinot et al. [33]	High	3	Low	1	4	Low	1	Low	1	2	Low	1	Low	1	2	Low	1	9	L
	Biais et al. [34]	High	3	Low	1	4	Low	1	Low	1	2	Low	1	Low	1	3	Low	1	10	M
	Biais et al. [35]	Unclear	2	Low	1	3	Low	1	Low	1	2	Low	1	Low	1	2	Low	1	8	L
	Guinot et al. [36]	Unclear	2	High	3	5	Low	1	Low	1	2	High	3	Low	1	4	Low	1	12	H
	Preisman et al. [37]	High	3	High	3	6	Low	1	Low	1	2	Low	1	Low	1	2	Low	1	11	H
	Biais et al. [38]	High	3	Low	1	4	Low	1	Low	1	2	Low	1	Low	1	2	High	3	11	H
	De Broca et al. [39]	Unclear	2	Low	1	3	Low	1	Low	1	2	Low	1	Low	1	2	Low	1	8	L
Intensive care unit	Wu et al. [40] Smorenberg et al. [41]	UncleaR	2	Low	1	3	Low	1	Low	1	2	Low	1	Low	1	2	Low	1	8	L
		Low	1	High	3	4	Low	1	Low	1	2	Low	1	Low	1	2	High	3	11	H
	Muller et al. [29]	Unclear	2	Low	1	3	Low	1	Low	1	2	Low	1	Low	1	2	Low	1	8	L
	Monge Garcia et al. [42]	Low	1	Unclear	2	3	Low	1	Unclear	2	3	High	3	Low	1	4	High	3	13	H
	Perel et al. [28]	High	3	Low	1	4	Low	1	Low	1	2	Low	1	Low	1	2	High	3	11	H
	Yonis et al. [43]	Unclear	2	High	3	5	Unclear	2	Low	1	3	Low	1	Low	1	2	Low	1	11	H
	Xiao-ting et al. [44]	Unclear	2	Low	1	3	Low	1	Low	1	2	Low	1	Low	1	2	High	3	10	H
	Mallat et al. [45]	High	3	Low	1	4	Low	1	Low	1	2	Low	1	Low	1	2	Low	1	9	M
	Georges et al. [46]	High	3	Low	1	4	Low	1	Low	1	2	Low	1	Low	1	2	Low	1	9	M
	Wilkman et al. [47]	High	3	Low	1	4	Low	1	Low	1	2	Low	1	Low	1	2	High	3	11	H
	Jozwiak et al. [48]	Unclear	2	Low	1	3	Low	1	Low	1	2	Low	1	Low	1	2	Low	1	8	L
	Monnet et al. [23]	High	3	Low	1	4	Low	1	Low	1	2	Low	1	Low	1	2	Low	1	9	M
	Myatra et al. [49]	Unclear	2	Low	1	3	Low	1	Low	1	2	Low	1	Low	1	2	Low	1	8	L
	Monnet et al. [50]	Unclear	2	Low	1	3	Low	1	Low	1	2	Low	1	Low	1	2	Low	1	8	L

For each study, the risk of bias is calculated as the sum of the four categories; we calculated the sum of these points. L = studies showing a score below the median of the sums of all studies. H = studies showing a score above the median of the sums of all studies. M = studies showing a score equal to the median of the sums of all studies

### Metanalysis of the included studies (see Figs. 2, 3, and 4)

The pooled AUC of the EEOT from two studies conducted in the OR [33, 34] and six [23, 43, 46, 48–50] in the ICU was 0.96 (95%CI 0.92–1.00). The pooled sensitivity of the test was 0.86 (95%CI 0.74–0.94), with *I*^2^ of 75% (95%CI 43–85%), and the pooled specificity was 0.91 (95%CI 0.85–0.95), with *I*^2^ of 35% (95%CI 0–69%). The median threshold identified was a 5% (4–8%) increase in the considered variable. The funnel plot of the included studies testing the EEOT shows a significant likelihood of publication bias (see Additional file 1: Figures S1 and S2).

**Fig. 2:**
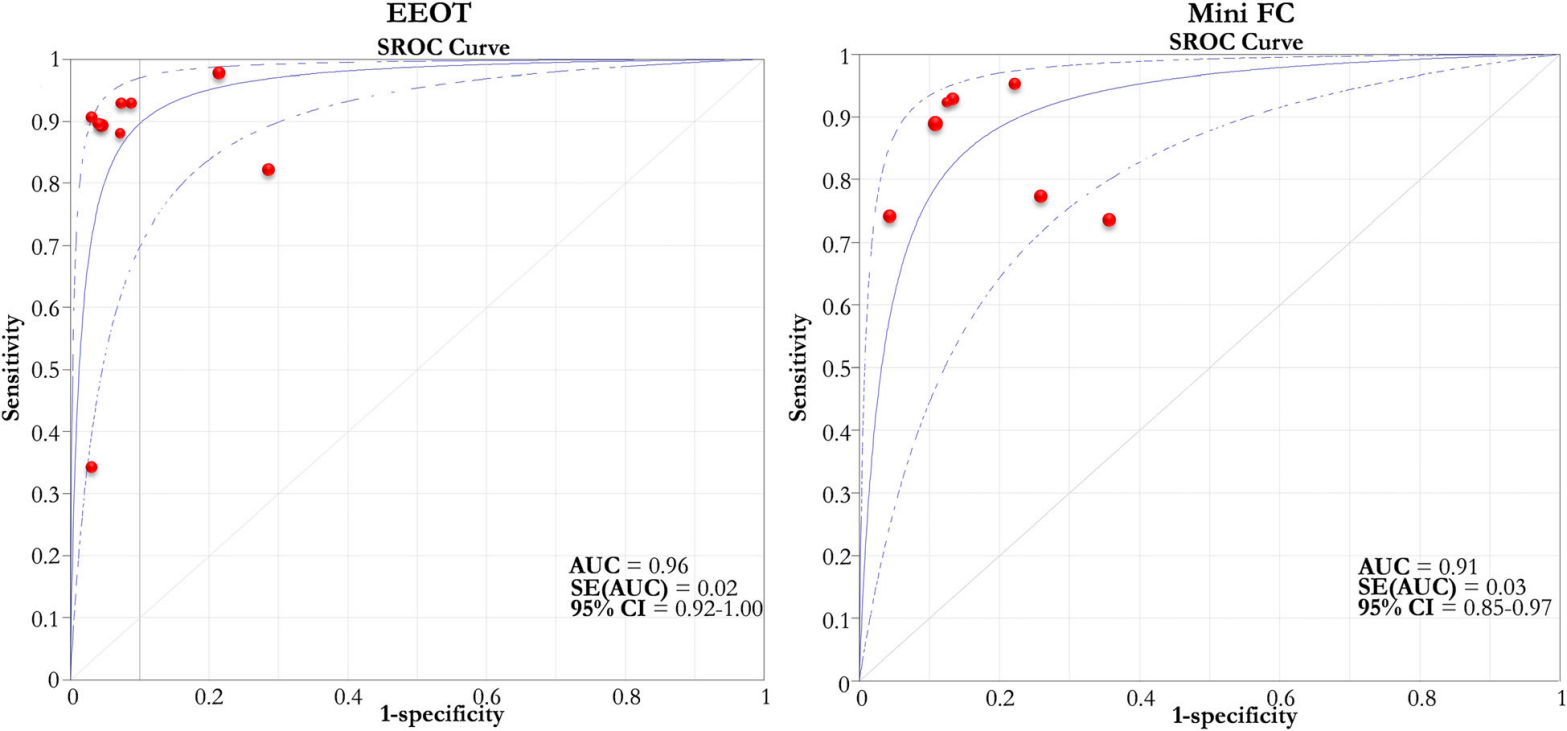
Pooled ROC curves of EEOT and mini-FC. Pooled receiver operating characteristic (ROC) curves of end-expiratory occlusion test (EEOT) [left panel, eight studies, area under the ROC curve = 0.96 (solid blue line) (95%CI 0.92–1.00; dashed blue lines)] and mini-fluid challenge (mini-FC) [right panel, seven studies, area under the ROC curve = 0.91 (solid blue line) (95%CI 0.85–0.97; dashed blue lines)] constructed by considering the hemodynamic effects of the EEOT or mini-FC on stroke volume or its surrogates and those induced by fluid challenge administration. Red circles represent each study included in the metanalysis and the size of each solid circle indicates the size of each study (software MetaDiSC®, version 1.4, see text and Table 3 for details)

**Fig. 3:**
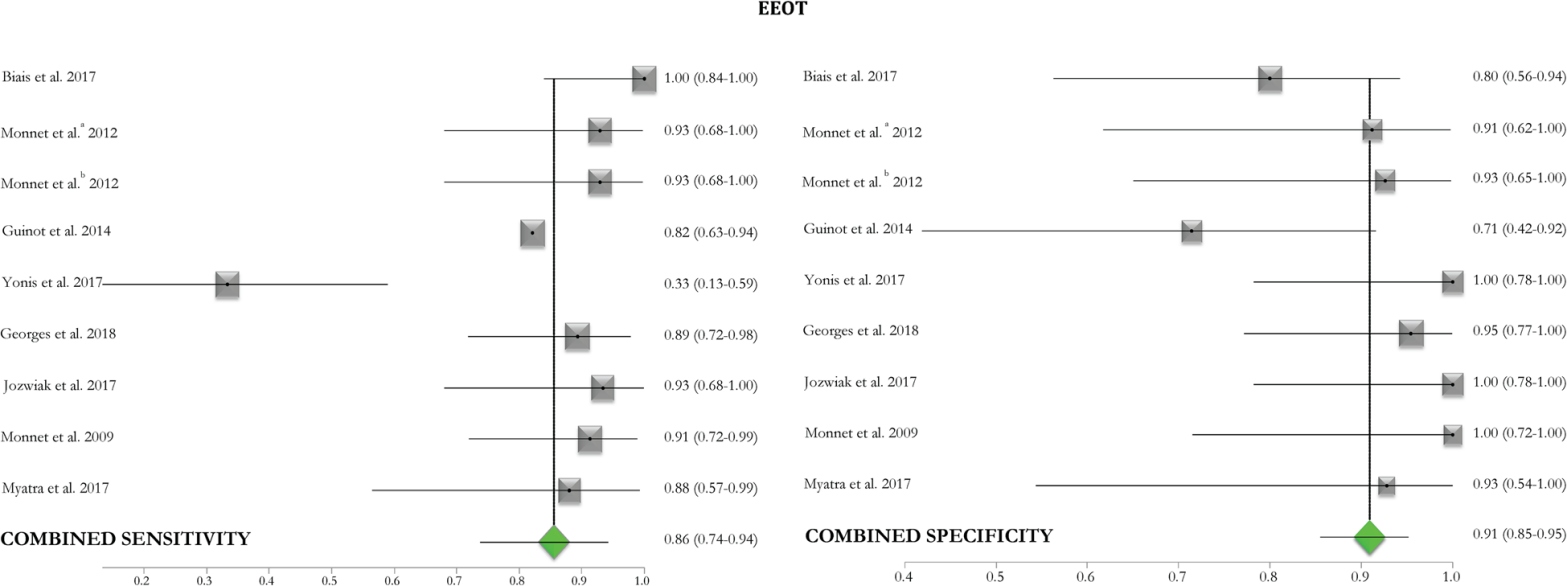
EEOT forest plot of included studies. Forest plot reporting the pooled sensitivity and specificity (green diamonds) of the end-expiratory occlusion test (EEOT) in predicting of fluid responsiveness by considering the changes in stroke volume or its surrogates after the test and those induced by fluid challenge administration. Black squares represent the values of sensitivity and specificity (with 95% confidence intervals; black lines) of each study included in the metanalysis, and the size of each square indicates the size of each study. The definitions Monnet et al. “a” and “b” refer to the two populations investigated in the study [50] (see also Table 3 and see text for details). 95%CI, 95% confidence intervals

**Fig. 4:**
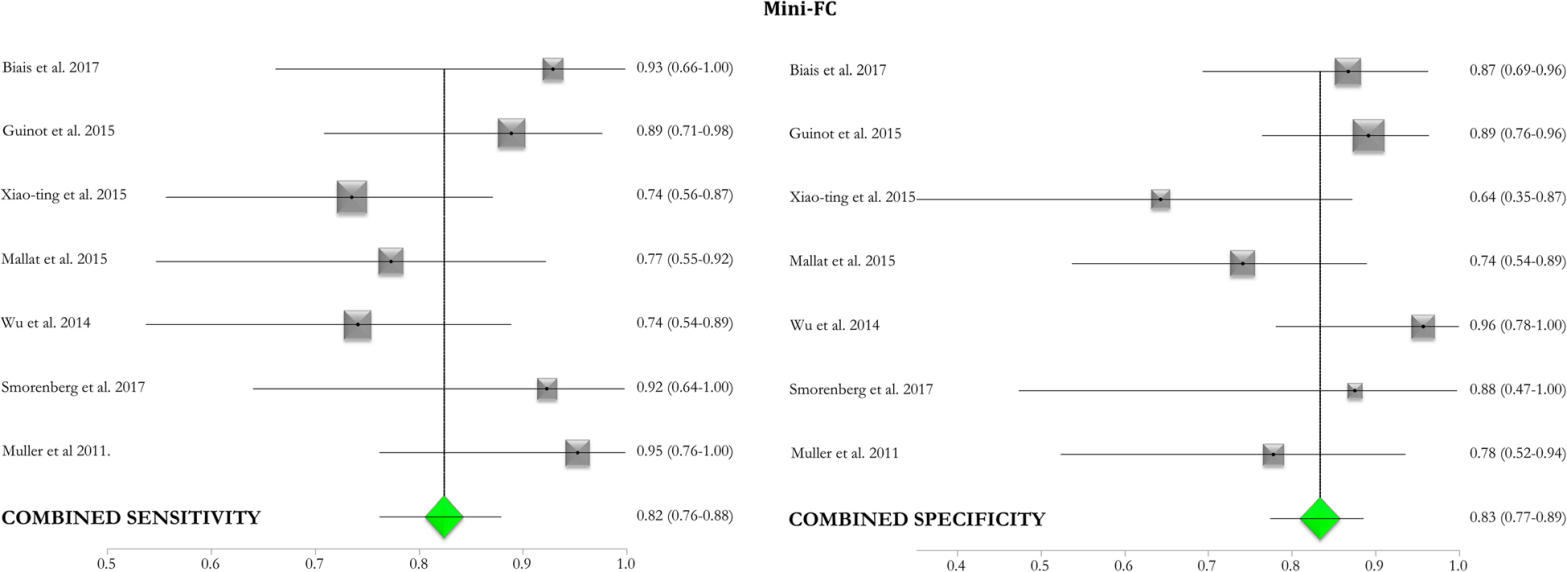
Mini-FC forest plot of included studies. Forest plot reporting the pooled sensitivity and specificity (green diamonds) of the mini-fluid challenge (mini-FC) in predicting fluid responsiveness by considering the changes in stroke volume or its surrogates after the test and those induced by fluid challenge administration. Black squares represent the values of sensitivity and specificity (with 95% confidence intervals; black lines) of each study included in the metanalysis, and the size of each square indicates the size of each study. 95%CI, 95% confidence intervals

The pooled AUC of the mini-FC obtained from two studies conducted in the OR [35, 36] and five [29, 40, 41, 44, 45] in the ICU was 0.91 (95%CI 0.85–0.97). The pooled sensitivity of the test was 0.82 (95%CI 0.76–0.88), with *I*^2^ of 26.9% (95%CI 0–69%), and pooled specificity was 0.83 (95%CI 0.77–0.89), with *I*^2^ of 34% (95%CI 0–71%). The median threshold identified was a 5% (3.0–7.0%) increase in the considered variable.

The funnel plot for the included studies testing the mini-FC shows a small likelihood of publication bias (see Additional file 1: Figures S3 and S4). Moreover, it was possible to calculate a pooled correlation of *r* = 0.68 (95%CI 0.41–0.84) between the changes in the cardiac flow parameters after mini-FC application and after FC administration from data obtained from 6 studies [29, 36, 40, 41, 44, 45].

## Discussion

The main findings of this systematic review conducted in ICU and OR patients are as follows: (1) the EEOT and the mini-FC have been tested in the OR and ICU and shown good sensitivity and specificity for predicting fluid responsiveness; (2) currently, the literature provides insufficient data regarding the other FHTs to assess a pooled quantification of their reliability in predicting fluid responsiveness; and (3) publication bias, small-sized study effects, and methodological heterogeneity of the individual studies should be considered.

### EEOT

Of the tests studied, the EEOT showed the highest sensitivity and specificity [pooled AUC of 0.96 (95%CI 0.92–1.00); pooled sensitivity and specificity of 0.86 (95%CI 0.74–0.94) and 0.91 (95%CI 0.85–0.95), respectively, with a best threshold of 5% (4.0–8.0%) of increase in SV or its surrogates; see Fig. 3 and Table 3]. In the two studies reporting an AUC higher than 0.90, the percentage of patients included in the gray zone was 17–20% [34, 46] (see Table 3).

**Table 3:** Reported ROC values of the included studies

	Studies	Functional hemodynamic test	Parameter	AUC (95%CI)	Sensitivity (%)	Specificity (%)	Y	+PV	−PV	+LR	−LR	Threshold	GZ high	GZ low	Pt in GZ (%)
Operating room	Guinot et al. [33]	EEOT: ventilation interrupted for 15 s	DELTA SV (%)	0.78 (0.63–0.89)	82	71	NA	85	67	2.87	0.25	2.3	NA	NA	NA
	Biais et al. [34]	EEOT: ventilation interrupted for 30 s	DELTA SV (%)	0.91 (0.81–1.0)	100	81	0.81	84	100	NA	NA	5	8	4	17
	Biais et al. [35]	Mini-FC: 100 ml in 120 s	DELTA SV (%)	0.95 (0.90–0.99)	93	85	0.78	NA	NA	NA	NA	6	7	4	19
	Guinot et al. [36]	Mini-FC: 100 ml in 60 s	DELTA SV (%)	0.93 (0.84–0.97)	89	89	NA	83	93	8.18	0.12	7	8	3	14
	Preisman et al. [37]	RSVT: decrease in SAP after successive incremental pressure-controlled breaths (10, 20, and 30 cmH_2_O)	RSVT (slope, mmHg/cmH_2_O)	0.96 (0.92–1.0)	93	89	NA	NA	NA	NA	NA	0.51	NA	NA	NA
	Biais et al. [38]	LRM: 30 cmH20/30 s	DELTA SV (%)	0.96 (0.81–0.99)	88	92	NA	NA	NA	NA	NA	30	− 22	− 37	36
	De Broca et al. [39]	LRM: 25 cmH20/25 s	DELTA SV (%)	0.95 (0.91–0.99)	92	96	0.92	100	89	NA	NA	16	− 15	− 17	8
Intensive care unit	Wu et al. [40]	Mini-FC: 50 ml in 10 s	DELTA VTI (%)	0.83 (0.69–0.96)	74	95	NA	94	79	NA	NA	9	NA	NA	NA
	Smorenberg et al. [41]	Mini-FC: 100 ml in 120 s	DELTA CO (%)	0.85 (0.63–0.97)	NA	NA	NA	92	88	NA	NA	2.3	NA	NA	NA
	Muller et al. [29]	Mini-FC: 100 ml in 60 s	DELTA VTI (%)	0.92 (0.78–0.98)	95	78	NA	NA	NA	NA	NA	3	NA	NA	NA
	Monge Garcia et al. [42]	Airway pressure elevated up to 30 cmH20 for 10 s	DELTA PP (%)	0.98 (0.84–0.99)	91	95	NA	91	95	NA	NA	52	NA	NA	NA
	Perel et al. [28]	RSVT: decrease in SAP after successive incremental pressure-controlled breaths (5,10, 15, and 20 cmH_2_O)	RSVT (slope, mmHg/cmH_2_O)	0.89 (0.72–1.0)	87.5	83	NA	NA	NA	NA	NA	0.24	NA	NA	NA
	Yonis et al. [43]	EEOT: ventilation interrupted for 15 s	DELTA CI (%)	0.65 (0.46–0.84)	33	100	NA	NA	NA	INF	0.67	10	11	− 4	79
	Yonis et al. [43]	Trendelemburg maneuver	DELTA CI (%)	0.9 (0.8–1.0)	87	89	NA	NA	NA	7.9	0.15	8	12	5	30
	Xiao-ting et al. [44]	Mini-FC: 100 ml in 60 s	DELTA CI (%)	0.83 (0.69–0.96)	73.2	60.6	NA	NA	NA	NA	NA	5.4	NA	NA	NA
	Mallat et al. [45]	Mini-FC: 100 ml in 60 s	DELTA CI (%)	0.78 (0.64–0.88)	77	74	NA	NA	NA	NA	NA	5.2	12.6	− 1.5	67
	Georges et al. [46]	EEOT: ventilation interrupted for 12 s	DELTA VTI (%)	0.96 ± 0.03	89	95	0.85	NA	NA	NA	NA	9	10	6	20
	Wilkman et al. [47]	PEEP elevated from 10 to 20 cmH_2_O for 60–120 s	DELTA MAP (%)	0.91 (0.77–1.0)	83	86	NA	71	92	NA	NA	− 10.2	NA	NA	NA
	Jozwiak et al. [48]	EEOT: ventilation interrupted for 15 s	DELTA CI (%)	0.98 (0.85–1.0)	93	100	0.93	NA	NA	NA	NA	4	NA	NA	NA
	Monnet et al. [23]	EEOT: ventilation interrupted for 15 s	DELTA CI (%)	0.97 (0.85–0.99)	91	100	NA	NA	NA	NA	NA	5	NA	NA	NA
	Myatra et al. [49]	*V*t raised up from 6 to 8 ml/kg for 1 min	DELTA PPV (%)	0.99 (0.98–1.0)	94	100	NA	100	93	NA	NA	3.5	NA	NA	NA
	Myatra et al. [49]	*V*t raised up from 6 to 8 ml/kg for 1 min	DELTA SVV (%)	0.97 (0.92–1.0)	88	100	NA	100	88	NA	NA	2.5	NA	NA	NA
	Myatra et al. [49]	EEOT: ventilation interrupted for 15 s (6 ml/kg ventilation)	DELTA CI (%)	0.44 (0.23–0.66)	NA	NA	NA	NA	NA	NA	NA	NA	NA	NA	NA
	Myatra et al. [49]	EEOT: ventilation interrupted for 15 s (8 ml/kg ventilation)	DELTA CI (%)	0.95 (0.88–1.0)	88	93	NA	93	78	NA	NA	4.1	NA	NA	NA
	Monnet et al. [50]	EEOT: ventilation interrupted for 15 s (Crs < 30 cmH_2_O/ml)	DELTA CI (%)	0.97 ± 0.03	93	91	0.85	93	91	NA	NA	5	NA	NA	NA
	Monnet et al. [50]	EEOT: ventilation interrupted for 15 s (Crs > 30 cmH_2_O/ml)	DELTA CI (%)	0.93 ± 0.05	93	92	0.84	93	92	NA	NA	5	NA	NA	NA

The area under the receiving operating characteristic (ROC) curve (AUC) of each study is reported as median (25th–75th interquartile) or mean (± standard deviation), as stated in the original article

*NA* data not available, *CI* cardiac index, *CO* cardiac output, *SV* stroke volume, *SVI* stroke volume index, *SVV* stroke volume variation, *PPV* pulse pressure variation, *VTI* velocity time integral, *RSVT* respiratory systolic variation test, *PP* pulse pressure, *MAP* mean arterial pressure, *SAP* systolic arterial pressure, *ND* not defined, *Vt* tidal volume, *Crs* respiratory compliance, *EEOT* end-expiratory occlusion test, *LRM* lung recruitment maneuver, *RSVT* respiratory systolic variation test, *PEEP* positive end-expiratory pressure, *FC* fluid challenge, *Y* Youden index, *+PV* positive predictive value, *−PV* negative predictive value, *+LR* positive likelihood ration, *−LR* negative likelihood ratio, *GZ* gray zone, *Pt* patients, *INF* infinite

This FHT was first proposed by Monnet et al. [23] and predicts fluid responsiveness by assessing changes in CO, or its surrogates, following a few second interruption to mechanical ventilation. In preload-dependent patients, this maneuver increases venous return and right ventricular and then subsequently left ventricular stroke volume. The potential drawbacks of this FHT include that it may be limited by patient positioning, the baseline tidal volume ventilation adopted, and the hemodynamic effects of residual spontaneous breathing efforts. Only one study used the EEOT to assess fluid responsiveness in prone ICU patients with moderate ARDS, reporting an AUC of 0.65 (0.46–0.84) [43]. Prone positioning affects the venous return by compressing the inferior cava vein and changing the intra-abdominal pressure [51–53], which may reduce the changes in CO and SV seen in response to the ventilatory challenge and limit the reliability of the EEOT.

The change in intrathoracic pressure may be insufficient to adequately increase right ventricular preload when a lung-protective ventilation strategy is used. Also, if the neural trigger for ventilation is preserved, a 15- to 30-s expiratory hold would result in a progressive increase in inspiratory pressure [54], affecting the venous return and the reliability of the FHT. Unfortunately, data regarding these issues is limited and contradictory.

In the OR, the EEOT performed better in a study using a mean tidal volume of 6.8 ml/kg [34], when compared to another study using 8.2 ml/kg [33]. In the ICU, the median tidal volume in those studies enrolling supine patients was 6.8 ml/kg (6.1–7.3). The EEOT failed to predict fluid responsiveness in the study of Myatra et al. using a 6-ml/kg ventilation [49], whereas Jozwiak et al. reported an AUC of 0.98 (0.85–1.0) using a 6.2-ml/kg ventilation. Interestingly, these two latter studies reported a comparable mean total respiratory system compliance in the enrolled patients (28 vs. 36 ml/cmH_2_O, respectively).

Monnet et al. reported an EEOT failure as high as 22.5%, due to the patient effort against an occluded airway [23]. However, none of the other studies using this FHT reported this failure rate. Four of the five studies reported no spontaneous breathing activity during assisted-controlled ventilation (see Table 1), implying the level of sedation was inhibiting neural triggering. None of these studies reported a flowchart showing the overall number of excluded patients, limiting the assessment of EEOT reliability during visible spontaneous breathing activity, which is a potential drawback for assessing fluid responsiveness.

### Mini-FC

The mini-FC showed a pooled AUC of 0.91 (95%CI 0.85–0.97). The pooled sensitivity and specificity were 0.82 (95%CI 0.76–0.88) and 0.83 (95%CI 0.77–0.89), respectively, with a best threshold of 5% (3.0–7.0%) increase in SV or its surrogates, see Fig. 4 and Table 3. These values of the pooled ROC curve imply a moderate overlap in the distribution of responders and non-responders.

In the two studies reporting an AUC higher than 0.90, the percentage of patients included in the gray zone was approximately 14–19% [35, 36] (see Table 3). Moreover, the performance of this FHT was comparable under stable conditions in the OR (using uncalibrated tools) and in more unstable ICU patients (using calibrated tools) (see Table 1).

The dose of the mini-FC was not fixed. Most of the studies used a bolus of 100 ml infused over 60 s, but Wu et al. demonstrated that a 10% of change in SV following the infusion of a 50-ml bolus in 10 s reliably predicted fluid responsiveness [40].

Some may argue that the mini-FC should not be considered an appropriate FHT, since the response to the first small aliquot of fluids is actually included into the response to the final volume administered, therefore not predicting the response to the whole FC, but only to a part of it. However, recent studies have shown different components of FC, related to the response (the extent of SV increase) and sustainability of the hemodynamic effect (the effect of SV over time) [55–57]. The mini-FC allows a dynamic evaluation of fluid administration, preventing inappropriate administration and allowing a tailored infusion. Moreover, this FHT has been also used in a different functional manner. In fact, Mallat et al. [45] demonstrated that a reduction in PPV [AUC = 0.92 (0.81–0.98)] or SVV [AUC = 0.91 (0.80–0.97)] following a mini-FC test was a better predictor of fluid responsiveness than an increase in CO. The cut-offs identified by the ROC curve for the changes in PPV and SVV are even smaller (2.0%) than the changes in CO (5.2%), implying a high precision of measurement, whichever hemodynamic tool is used.

### Other FTHs

All the other FHTs reported in the literature affect both right ventricular preload and afterload, by briefly altering intrathoracic pressure and, as a consequence, venous return and pulmonary vascular resistance.

The RSVT is based on the delivery of consecutive pressure-controlled inspiratory breaths, using incremental peak inspiratory pressures (up to 30 cmH_2_O) and plotting the minimal values of the systolic arterial pressure recorded after each breath against the related airway pressures (offline slope calculation) [28, 37]. Despite promising results obtained in both the OR and ICU [28, 37], the integration of respiratory and hemodynamic signals required to allow an online computation of the RSVT has never been achieved at the bedside.

Raising intrathoracic pressure by increasing peak inspiratory pressure using either a Valsalva maneuver [42]or the end-expiratory occlusion pressure [47] or by performing a LRM are all FHTs that induce a sudden change in right ventricular preload and afterload. LRMs have been successfully applied in the OR, showing a comparable AUC in neurosurgery [38] and general abdominal surgery [39]. However, Biais et al. found that the best threshold to define fluid responsiveness was a 30% reduction in SV, but De Broca et al. showed only a 16% reduction was required [39], suggesting caution in the interpretation on this FHT.

Finally, more recently, Myatra et al. successfully enhanced the reliability of baseline indexes of fluid responsiveness by increasing the tidal volume from 6 to 8 ml/kg for 1 min (the tidal volume challenge) [49]. This simple and quick FHT could be used in patients undergoing protective ventilation but should be tested in larger ICU populations both with and without spontaneous breathing activity.

### Bedside application

**Fig. 5:**
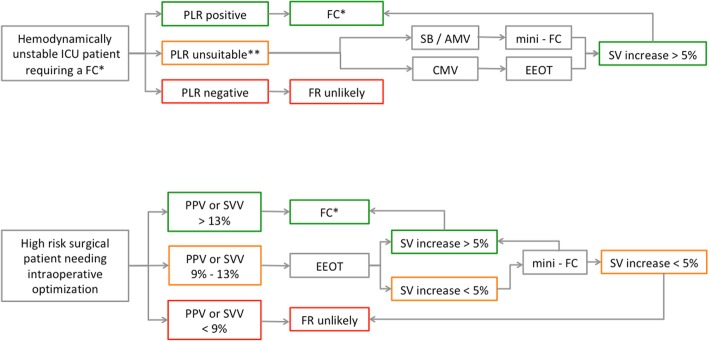
Clinical algorithm for EEOT and mini-FC application in the ICU and the OR. In the OR, FHTs can be added to the dynamic indexes evaluation, considering the gray zone reported in the literature [21]. When PPV or SVV values range within the gray zone, we suggest the use of the EEOT, as the first step. A clear positive response (SV increase > 5%) suggests fluid responsiveness, whereas a negative/uncertain response could be further investigated by the consequent use of the mini-FC, as indicated. In critically ill patients, the need of FC administration is often evaluated combining different signs and measurements [58]. In this context, the EEOT (in patient undergoing controlled mechanical ventilation) and the mini-FC (in patients retaining to some extent a spontaneous breathing effort) can be useful when the PLR is unsuitable.*We suggest a FC of 4 ml/kg [55] over 10 min. **Intra-abdominal hypertension; uncontrolled pain, cough, discomfort, and awakening; hip/leg fractures; uncontrolled intracranial hypertension. ICU, intensive care unit; OR, operating room; FC, fluid challenge; PLR, passive leg raising; CMV, controlled mechanical ventilation; SB, spontaneously breathing patients; AMV, assisted mechanical ventilation; PPV, pulse pressure variation; SVV, stroke volume variation; EEOT, end-expiratory occlusion test; SV, stroke volume

The EEOT and the mini-FC could be appropriately used in different clinical scenarios, especially when the PLR is unsuitable or in adjunct to that. In Fig. 5, we propose a step-by-step clinical algorithm in patients who would benefit from FC administration in the OR and the ICU.

### Limitations

The comparability of the included studies is limited by the heterogeneity of FC administration used as the reference point (see Table 1). Aya et al. have previously demonstrated that a FC should be at least 4 ml/kg [55]. For this reason, some patients enrolled in those studies adopting a smaller dose of FC (3.7 ml/kg [34]; 3.3 ml/kg [35, 38]) may be underchallenged, which may have affected the observed rate of fluid responsiveness and, in turn, the ROC curve construction.

Another potential source of bias is related to the different hemodynamic tools used to assess both fluid responsiveness and FHT reliability. In fact, when considering the median cutoff value identifying responders from non-responders (about 5% for both the EEOT and the mini-FC), the accuracy of measurement of the changes in CO, or its surrogates, is of pivotal importance. For example, the negative results of Guinot et al. [33], conducted in the OR, have been questioned as the esophageal Doppler does not measure the change in aortic diameter and could therefore underestimate the change in SV during either the EEOT or the FC [59].

Additionally, the reliability of different calibrated and uncalibrated tools in tracking the dynamic trends of CO may not be consistent and may be below the boundaries of the accuracy and precision of the Critchley-Critchley criteria [60, 61]. For instance, the reproducibility of the measurements obtained by the different hemodynamic tools has never been reported in the included studies. This implies that small changes in CO, or its surrogates, after a FHT may be inaccurately detected in the OR, where the hemodynamic monitoring is usually performed with uncalibrated tools, whereas the use of calibrated techniques by means of thermodilution could reduce the risk of imprecise measurements in ICU.

All the included studies had a small-sized single-center design and enrolled a median number of patients rather small [39 (IQR 25–50)], and about 43% of the included studies were classified in the subgroup with the highest risk of bias, mainly because of the drawbacks related to the patient selection, according to the QUADAS-2 score (see Table 2). This limitation along with the use of different cutoff values, thresholds, and measurement techniques to assess fluid responsiveness potentially produced heterogeneity in the response to the FC administration. As confirmed, the proportion of responders ranged between 30 and 71% across the included studies. The bedside application is also limited in those potentially misclassified patients (roughly 20% in the reported studies) included in the gray zone of the ROC curve, where the predictive power of the FHT is rather low. Another source of heterogeneity may be related to the different sample sizes of the included studies, as confirmed by the large interquartile ranges of the *I*^2^. Finally, we did not include non-full-text studies, studies not in English, and unpublished studies, and this systematic review was not prospectively registered in PROSPERO, an international database of systematic reviews in health and social care, increasing the overall risk of reporting bias.

For all these key aforementioned reasons, despite the increasing number of studies in this field, the clinical applicability and utility of the FTHs should be assessed by a large multicentric trial. Although pooling a few data from studies carried out in different settings could bias the interpretation of the findings, the identification of the current evidence, associated to a careful assessment of the confounding factors, could help in designing future studies.

## Conclusions

Both the EEOT and the mini-FC showed good sensitivity and specificity in predicting fluid responsiveness in the OR and ICU. The different methods of FC administration used as the reference standard and the different hemodynamic tools used to track hemodynamic changes with each FHT limit the comparability of the studies. Other promising FHTs should be tested in larger populations.

## Additional files

Additional file 1: **Table S1.** Definition of potential bias for the enrolled studies. **Table S2.** Baseline hemodynamic parameters before FHT application in responders and non-responders. **Figure S1.** Funnel plot for publication bias analysis of sensitivity of the end-expiratory occlusion test (EEOT). **Figure S2.** Funnel plot for publication bias analysis of specificity of the end-expiratory occlusion test (EEOT). **Figure S3.** Funnel plot for publication bias analysis of sensitivity of the mini-fluid challenge test (mini-FC). **Figure S4.** Funnel plot for publication bias analysis of specificity of the mini-fluid challenge test (Mini-FC) (DOCX 3448 kb)

## Data Availability

The datasets used and/or analyzed during the current study are available from the corresponding author on reasonable request.

## Abbreviations

AUC: Area under the ROC curve
CI: Cardiac index
EEO: End-expiratory occlusion test
FC: Fluid challenge
FHT: Functional hemodynamic test
ICU: Intensive care unit
IQR: 25th–75th interquartile range
LRM: Lung recruitment maneuver
OR: Operating room
PLR: Passive leg raising
PPV: pulse pressure variation
ROC: Receiver operating characteristic
RSVT: Respiratory systolic variation test
SD: Standard deviation
SV: Stroke volume
SVI: Stroke volume index
SVV: Stroke pressure variation

## References

[1] Cecconi M, De Backer D, Antonelli M, Beale R, Bakker J, Hofer C (2014). Consensus on circulatory shock and hemodynamic monitoring. Task Force of the European Society of Intensive Care Medicine. Intensive Care Med.

[2] Holte K, Kehlet H (2006). Fluid therapy and surgical outcomes in elective surgery: a need for reassessment in fast-track surgery. J Am Coll Surg.

[3] Boyd JH, Forbes J, Nakada TA, Walley KR, Russell JA (2011). Fluid resuscitation in septic shock: a positive fluid balance and elevated central venous pressure are associated with increased mortality. Crit Care Med.

[4] National Heart L, Wiedemann HP, Wheeler AP, Bernard GR, Thompson BT, Blood Institute Acute Respiratory Distress Syndrome Clinical Trials N (2006). Comparison of two fluid-management strategies in acute lung injury. NEJM.

[5] Cecconi M, Corredor C, Arulkumaran N, Abuella G, Ball J, Grounds RM (2013). Clinical review: goal-directed therapy-what is the evidence in surgical patients? The effect on different risk groups. Crit care.

[6] Hamilton MA, Cecconi M, Rhodes A (2011). A systematic review and meta-analysis on the use of preemptive hemodynamic intervention to improve postoperative outcomes in moderate and high-risk surgical patients. Anesth Analg.

[7] Lobo SM, de Oliveira NE (2013). Clinical review: what are the best hemodynamic targets for noncardiac surgical patients?. Crit Care.

[8] Marik PE (2014). Perioperative hemodynamic optimization: a revised approach. J Clin Anesth.

[9] Voldby AW, Brandstrup B (2016). Fluid therapy in the perioperative setting-a clinical review. J Intensive Care.

[10] Thacker JK, Mountford WK, Ernst FR, Krukas MR, Mythen MM (2016). Perioperative fluid utilization variability and association with outcomes: considerations for enhanced recovery efforts in sample us surgical populations. Ann Surg.

[11] Cecconi M, Hofer C, Teboul JL, Pettila V, Wilkman E, Molnar Z (2015). Fluid challenges in intensive care: the FENICE study: a global inception cohort study. Intensive Care Med.

[12] Cecconi M, Parsons AK, Rhodes A (2011). What is a fluid challenge?. Curr Opin Crit Care.

[13] Vincent JL, Weil MH (2006). Fluid challenge revisited. Crit Care Med.

[14] Messina A, Longhini F, Coppo C, Pagni A, Lungu R, Ronco C (2017). Use of the fluid challenge in critically ill adult patients: a systematic review. Anesth Analg.

[15] Messina A, Pelaia C, Bruni A, Garofalo E, Bonicolini E, Longhini F (2018). Fluid challenge during anesthesia: a systematic review and meta-analysis. Anesth Analg.

[16] Vincent JL (2011). “Let’s give some fluid and see what happens” versus the “mini-fluid challenge”. Anesthesiology.

[17] Pinsky MR (2015). Functional hemodynamic monitoring. Crit Care Clin.

[18] Pinsky MR, Payen D (2005). Functional hemodynamic monitoring. Crit Care.

[19] Biais M, Ehrmann S, Mari A, Conte B, Mahjoub Y, Desebbe O (2014). Clinical relevance of pulse pressure variations for predicting fluid responsiveness in mechanically ventilated intensive care unit patients: the grey zone approach. Crit Care.

[20] Mahjoub Y, Lejeune V, Muller L, Perbet S, Zieleskiewicz L, Bart F (2014). Evaluation of pulse pressure variation validity criteria in critically ill patients: a prospective observational multicentre point-prevalence study. Br J Anaesth.

[21] Cannesson M, Le Manach Y, Hofer CK, Goarin JP, Lehot JJ, Vallet B (2011). Assessing the diagnostic accuracy of pulse pressure variations for the prediction of fluid responsiveness: a “gray zone” approach. Anesthesiology.

[22] Hadian M, Pinsky MR (2007). Functional hemodynamic monitoring. Curr Opin Crit Care.

[23] Monnet X, Osman D, Ridel C, Lamia B, Richard C, Teboul JL (2009). Predicting volume responsiveness by using the end-expiratory occlusion in mechanically ventilated intensive care unit patients. Crit Care Med.

[24] Cherpanath TG, Hirsch A, Geerts BF, Lagrand WK, Leeflang MM, Schultz MJ (2016). Predicting fluid responsiveness by passive leg raising: a systematic review and meta-analysis of 23 clinical trials. Crit Care Med.

[25] Monnet X, Marik P, Teboul JL (2016). Passive leg raising for predicting fluid responsiveness: a systematic review and meta-analysis. Intensive Care Med.

[26] Cavallaro F, Sandroni C, Marano C, La Torre G, Mannocci A, De Waure C (2010). Diagnostic accuracy of passive leg raising for prediction of fluid responsiveness in adults: systematic review and meta-analysis of clinical studies. Intensive Care Med.

[27] Monnet X, Teboul JL (2015). Passive leg raising: five rules, not a drop of fluid!. Crit Care.

[28] Perel A, Minkovich L, Preisman S, Abiad M, Segal E, Coriat P (2005). Assessing fluid-responsiveness by a standardized ventilatory maneuver: the respiratory systolic variation test. Anesth Analg.

[29] Muller L, Toumi M, Bousquet PJ, Riu-Poulenc B, Louart G, Candela D (2011). An increase in aortic blood flow after an infusion of 100 ml colloid over 1 minute can predict fluid responsiveness: the mini-fluid challenge study. Anesthesiology.

[30] Marik PE (2015). Fluid therapy in 2015 and beyond: the mini-fluid challenge and mini-fluid bolus approach. Br J Anaesth.

[31] Marik PE, Cavallazzi R, Vasu T, Hirani A (2009). Dynamic changes in arterial waveform derived variables and fluid responsiveness in mechanically ventilated patients: a systematic review of the literature. Crit Care Med.

[32] Whiting PF, Rutjes AW, Westwood ME, Mallett S, Deeks JJ, Reitsma JB (2011). Quadas-2: a revised tool for the quality assessment of diagnostic accuracy studies. Ann Intern Med.

[33] Guinot PG, Godart J, de Broca B, Bernard E, Lorne E, Dupont H (2014). End-expiratory occlusion manoeuvre does not accurately predict fluid responsiveness in the operating theatre. Br J Anaesth.

[34] Biais M, Larghi M, Henriot J, de Courson H, Sesay M, Nouette-Gaulain K (2017). End-expiratory occlusion test predicts fluid responsiveness in patients with protective ventilation in the operating room. Anesth Analg.

[35] Biais M, de Courson H, Lanchon R, Pereira B, Bardonneau G, Griton M (2017). Mini-fluid challenge of 100 ml of crystalloid predicts fluid responsiveness in the operating room. Anesthesiology.

[36] Guinot PG, Bernard E, Defrancq F, Petiot S, Majoub Y, Dupont H (2014). Mini-fluid challenge predicts fluid responsiveness during spontaneous breathing under spinal anaesthesia: an observational study. Eur J Anaesthesiol.

[37] Preisman S, Kogan S, Berkenstadt H, Perel A (2005). Predicting fluid responsiveness in patients undergoing cardiac surgery: functional haemodynamic parameters including the respiratory systolic variation test and static preload indicators. Br J Anaesth.

[38] Biais M, Lanchon R, Sesay M, Le Gall L, Pereira B, Futier E (2017). Changes in stroke volume induced by lung recruitment maneuver predict fluid responsiveness in mechanically ventilated patients in the operating room. Anesthesiology.

[39] De Broca B, Garnier J, Fischer MO, Archange T, Marc J, Abou-Arab O (2016). Stroke volume changes induced by a recruitment maneuver predict fluid responsiveness in patients with protective ventilation in the operating theater. Medicine (Baltimore).

[40] Wu Y, Zhou S, Zhou Z, Liu B (2014). A 10-second fluid challenge guided by transthoracic echocardiography can predict fluid responsiveness. Crit Care.

[41] Smorenberg A, Cherpanath TGV, Geerts BF, de Wilde RBP, Jansen JRC, Maas JJ (2018). A mini-fluid challenge of 150ml predicts fluid responsiveness using modelflow(r) pulse contour cardiac output directly after cardiac surgery. J Clin Anesth.

[42] Monge Garcia MI, Gil Cano A, Diaz Monrove JC (2009). Arterial pressure changes during the valsalva maneuver to predict fluid responsiveness in spontaneously breathing patients. Intensive Care Med.

[43] Yonis H, Bitker L, Aublanc M, Perinel Ragey S, Riad Z, Lissonde F (2017). Change in cardiac output during Trendelenburg maneuver is a reliable predictor of fluid responsiveness in patients with acute respiratory distress syndrome in the prone position under protective ventilation. Crit Care.

[44] Xiao-ting W, Hua Z, Da-wei L, Hong-min Z, Huai-wu H, Yun L (2015). Changes in end-tidal co2 could predict fluid responsiveness in the passive leg raising test but not in the mini-fluid challenge test: a prospective and observational study. J Crit Care.

[45] Mallat J, Meddour M, Durville E, Lemyze M, Pepy F, Temime J (2015). Decrease in pulse pressure and stroke volume variations after mini-fluid challenge accurately predicts fluid responsiveness. Br J Anaesth.

[46] Georges D, de Courson H, Lanchon R, Sesay M, Nouette-Gaulain K, Biais M (2018). End-expiratory occlusion maneuver to predict fluid responsiveness in the intensive care unit: an echocardiographic study. Crit Care.

[47] Wilkman E, Kuitunen A, Pettila V, Varpula M (2014). Fluid responsiveness predicted by elevation of peep in patients with septic shock. Acta Anaesthesiol Scand.

[48] Jozwiak M, Depret F, Teboul JL, Alphonsine JE, Lai C, Richard C (2017). Predicting fluid responsiveness in critically ill patients by using combined end-expiratory and end-inspiratory occlusions with echocardiography. Crit Care Med.

[49] Myatra SN, Prabu NR, Divatia JV, Monnet X, Kulkarni AP, Teboul JL (2017). The changes in pulse pressure variation or stroke volume variation after a “tidal volume challenge” reliably predict fluid responsiveness during low tidal volume ventilation. Crit Care Med.

[50] Monnet X, Bleibtreu A, Ferre A, Dres M, Gharbi R, Richard C (2012). Passive leg-raising and end-expiratory occlusion tests perform better than pulse pressure variation in patients with low respiratory system compliance. Crit Care Med.

[51] Edgcombe H, Carter K, Yarrow S (2008). Anaesthesia in the prone position. Br J Anaesth.

[52] Sudheer PS, Logan SW, Ateleanu B, Hall JE (2006). Haemodynamic effects of the prone position: a comparison of propofol total intravenous and inhalation anaesthesia. Anaesthesia.

[53] Wadsworth R, Anderton JM, Vohra A (1996). The effect of four different surgical prone positions on cardiovascular parameters in healthy volunteers. Anaesthesia.

[54] Truwit JD, Marini JJ (1992). Validation of a technique to assess maximal inspiratory pressure in poorly cooperative patients. Chest.

[55] Aya HD, Ster IC, Fletcher N, Grounds RM, Rhodes A, Cecconi M (2016). Pharmacodynamic analysis of a fluid challenge. Crit Care Med.

[56] Toscani L, Aya HD, Antonakaki D, Bastoni D, Watson X, Arulkumaran N (2017). What is the impact of the fluid challenge technique on diagnosis of fluid responsiveness? A systematic review and meta-analysis. Crit Care.

[57] Aya HD, Rhodes A, Chis Ster I, Fletcher N, Grounds RM, Cecconi M (2017). Hemodynamic effect of different doses of fluids for a fluid challenge: a quasi-randomized controlled study. Crit Care Med.

[58] Messina A, Greco M, Cecconi M (2019). What should I use next if clinical evaluation and echocardiographic haemodynamic assessment is not enough?. Curr Opin Crit Care.

[59] Monnet X, Teboul JL (2015). End-expiratory occlusion test: please use the appropriate tools!. Br J Anaesth.

[60] Critchley LA, Critchley JA (1999). A meta-analysis of studies using bias and precision statistics to compare cardiac output measurement techniques. J Clin Monit Comput.

[61] Hadian M, Kim HK, Severyn DA, Pinsky MR (2010). Cross-comparison of cardiac output trending accuracy of lidco, picco, flotrac and pulmonary artery catheters. Crit Care.

