# Supplementary material for: Functional hemodynamic tests: a systematic review and a metanalysis on the reliability of the end-expiratory occlusion test and of the mini-fluid challenge in predicting fluid responsiveness
Source: Crit Care. 2019 Jul 29;23:264. doi: 10.1186/s13054-019-2545-z (PMC6664788; doi:10.1186/s13054-019-2545-z)

**Functional hemodynamic assessment in critically ill and surgical patients. A systematic review of the available tests and a metanalysis on the reliability of the end-expiratory occlusion test and of the mini fluid challenge in predicting fluid responsiveness.**

Short Title: functional hemodynamic tests in intensive care and operating room.

Antonio Messina, MD, PhD^1^; Antonio Dell’Anna, MD^2,3^; Marta Baggiani; MD^4^; Flavia Torrini, MD^2,3^; Gianmarco Maresca, MD^2,3^; Victoria Bennett, MBBS, FFICM^5^; Laura Saderi, BSc^6^; Giovanni Sotgiu, MD, FERS^6^; Massimo Antonelli, MD^2,3^; Maurizio Cecconi, MD, FRCA, FFICM^1,7^

^1^Humanitas Clinical and Research Center – IRCCS, Rozzano – Milano, Italy; ^2^Department of Anesthesiology and Intensive Care Medicine, Catholic University of the Sacred Heart, Fondazione “Policlinico Universitario A. Gemelli”, Rome; ^3^Fondazione Policlinico Universitario A. Gemelli IRCCS, Rome, Italy; ^4^Department of Anesthesiology and Intensive Care Medicine, A.O.U. Maggiore della Carità, Novara, Italy; ^5^Department of Intensive Care Medicine, St George’s University Hospital NHS Foundation Trust, London, UK; ^6^Clinical Epidemiology and Medical Statistics Unit, Dept of Biomedical Sciences, University of Sassari, Research, Medical Education and Professional Development Unit, AOU Sassari, Sassari, Italy;^7^Humanitas University, Department of Biomedical Sciences, Pieve Emanuele – Milano, Italy.

**Supplementary Material**

Table S1. Definition of potential bias for the enrolled studies.

|  |  | **CRITERIA** | **LOW RISK** | **UNCLEAR RISK** | **HIGH RISK** |
| --- | --- | --- | --- | --- | --- |
| Patient selection | Risk of Bias | Patients were consecutively included | Consecutive inclusion stated | Period of enrolment indicated and consecutive inclusion not indicated | Non consecutive inclusion stated and no period of enrolment indicated |
|  |  | Inappropriate exclusions avoided | No inappropriate exclusions | NA | Inappropriate exclusions |
|  | Applicability judgments | Are there concerns that the included patients do not match the review question? | All the others | Spontaneously  breathing patients. | Spinal anesthesia or cardiac surgery; prone patients^1^ |
| Index test | Risk of Bias | Threshold used to define volume responsiveness pre-specified | Defined | NA | Undefined |
|  | Applicability judgments | Are there concerns that the included patients do not match the review question? | All the others | Spontaneously  breathing patients | Spinal anesthesia; cardiac surgery; prone patients^1^ |
| Reference Standard | Risk of Bias | The result of volume expansion on CO or SV assessed without knowledge of the result of the adopted FHT. | Assessed without knowledge of the result | NA | Assessed with knowledge of the result |
|  |  | The reliability of the device used in the study widely validated | OR: echocardiography by experts; PAC; calibrated tool, uncalibrated tool.  ICU: echocardiography by experts; PAC; calibrated tool, | OR: NA  ICU: uncalibrated tool; Bioimpedance - Bioreactance for postoperative patients | OR: Bioimpedance - Bioreactance  ICU: uncalibrated tool; Bioimpedance - Bioreactance for critically ill patients. |
|  | Applicability judgments | Are there concerns that the target condition as defined by the reference standard does not match the review question? | NO | NA | YES |
| Flow and Timing | Risk of Bias | An appropriate interval between the FHT and volume expansion | ≤ 5 minutes | NA | > 5 minutes or undefined |
|  |  | Patients received the same volume expansion | Same volume expansion | NA | Different volume expansion |
|  |  | All patients were included in the analysis. | All included | NA | Not all included |

The risk of bias of the enrolled studies was defined by The QUADAS 2 score, by two experts. For each domain the risk was judged as “low,” “high,” or “unclear.” If the answers to all signaling questions for a domain were “yes,” then risk of bias was judged low. If any signaling question was answered “no,” then potential bias was assessed by used the reported criteria. The cumulative risk of bias of each criterion was defined by the highest risk of bias reported. CO, cardiac output; SV, stroke volume; NA, not applicable; OR, operating room; PAC, pulmonary artery catheter; FHT, functional hemodynamic test.

^1^The highest risk of bias was decided considering the risk of 1) unpredictable changes in the ventilator pattern of spontaneously breathing patients or patients undergoing spinal anesthesia during the FHT; 2) the impairment of cardiac function after the surgery; 3) hemodynamic changes associated to prone positioning.

Table S2. Baseline hemodynamic parameters before FHT application in responders and non-responders.

|  | Operating Room | | | | |
| --- | --- | --- | --- | --- | --- |
|  | MAP  (mmHg) | HR  (beats/minute) | CO  (L/min) | SV  (ml/beat) | PPV  (%) |
| Responders | 71 (69 – 73) | 108 (100 – 116) | 4.2 (3.9 – 5.0) | 67 (64 - 70) | 14 (11 – 17) |
| Non-responders | 71 (68 – 77) | 111 (103 – 119) | 4.9 (4.5 – 7.1) | 77 (67 - 103) | 6 (5 – 9) |
| p value R vs. NR | 0.88 | 0.55 | 0.33 | 0.11 | 0.08 |
|  | Intensive Care Unit | | | | |
|  | MAP  (mmHg) | HR  (beats/minute) | CI  (L/min/m^2^) | SVI  (ml /beat/m^2^) | PPV  (%) |
| Responders | 78 (70 – 82) | 95 (80 – 106) | 2.7 (2.5 – 3.2) | 31 (26 - 37) | 10 (8 – 18) |
| Non-responders | 80 (72 – 84) | 91 (83 – 102) | 3.2 (2.7 – 3.7) | 32 (31 - 38) | 7 (6 – 9) |
| p value R vs. NR | 0.42 | 0.99 | 0.09 | 0.62 | 0.05 |

R, responders, NR, non-responders; OR, operating room; ICU, intensive care unit; MAP, mean arterial pressure; HR, heart rate; CO, cardiac output; SV, stroke volume; CI, cardiac index; SVI, stroke volume index; PPV, pulse pressure variation.

Figure S1: Funnel Plot for Publication Bias Analysis of sensitivity of the end-expiratory occlusion test (EEOT)


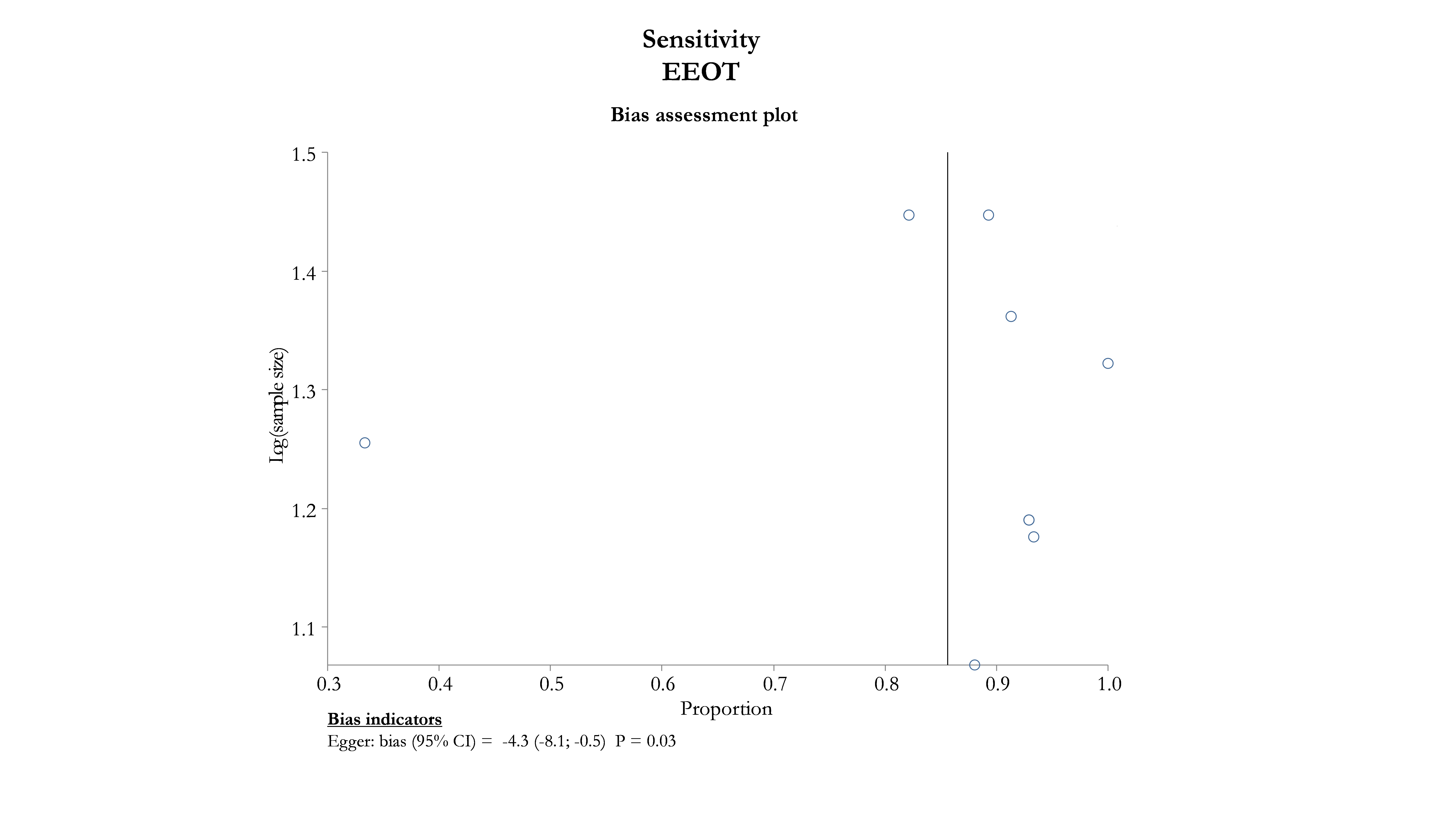


Figure S2: Funnel Plot for Publication Bias Analysis of specificity of the end-expiratory occlusion test (EEOT)


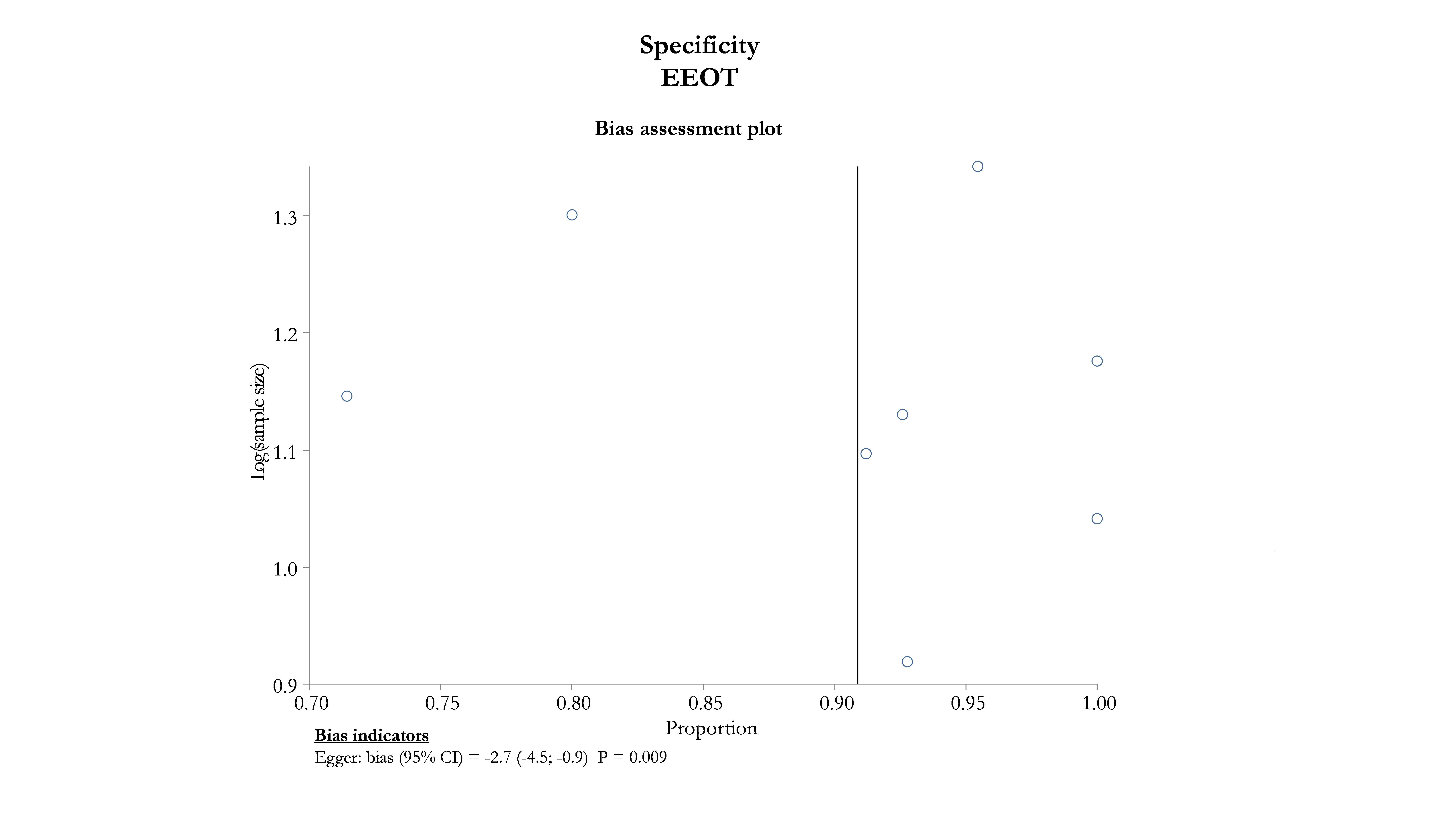


Figure S3: Funnel Plot for Publication Bias Analysis of sensitivity of the mini fluid challenge test (Mini FC)


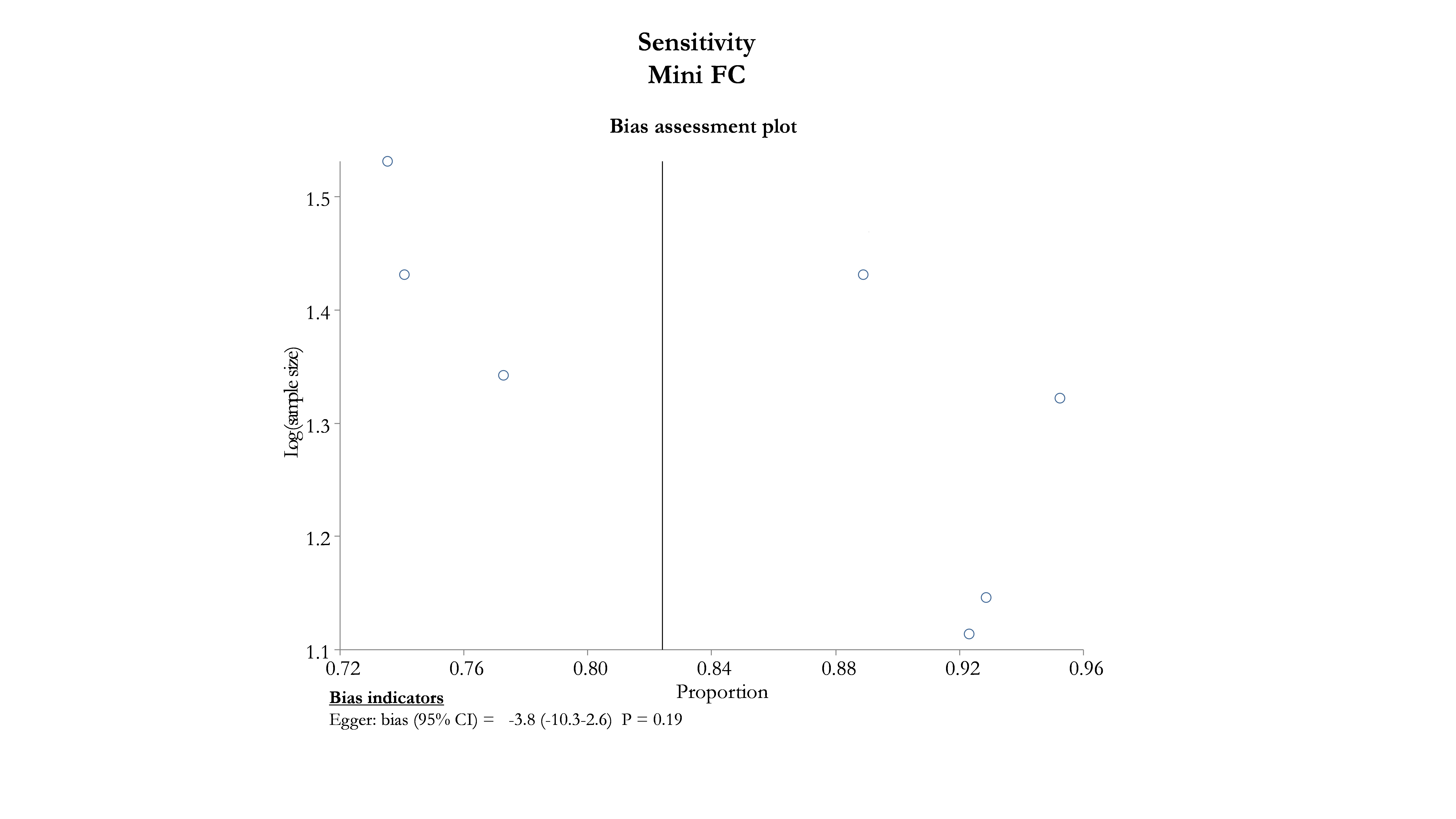


Figure S4: Funnel Plot for Publication Bias Analysis of specificity of the mini fluid challenge test (Mini FC)


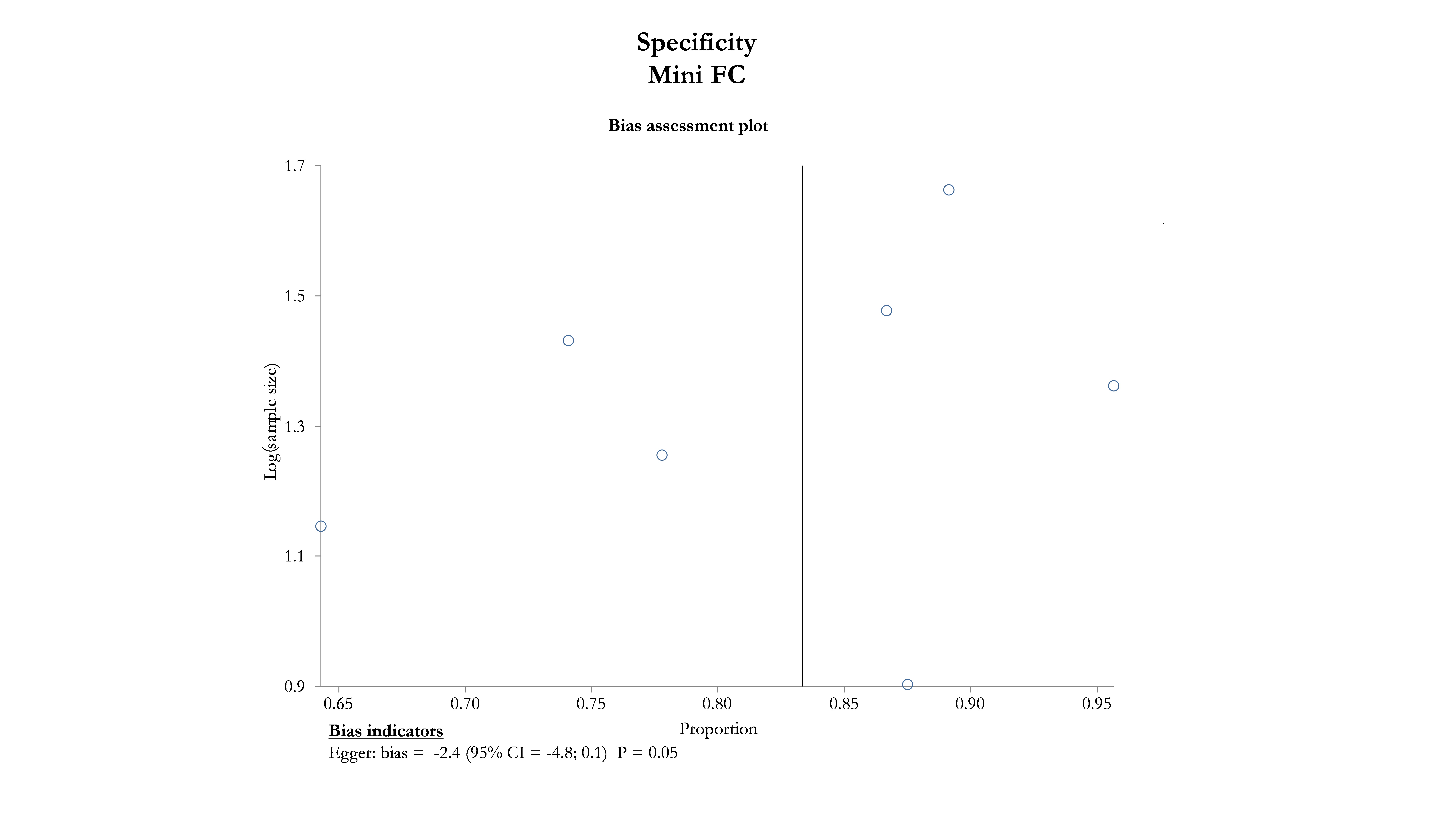

Supplement: Supplementary file 1 — Table S1. Definition of potential bias for the enrolled studies. Table S2. Baseline hemodynamic parameters before FHT application in responders and non-responders. Figure S1. Funnel plot for publication bias analysis of sensitivity of the end-expiratory occlusion test (EEOT). Figure S2. Funnel plot for publication bias analysis of specificity of the end-expiratory occlusion test (EEOT). Figure S3. Funnel plot for publication bias analysis of sensitivity of the mini-fluid challenge test (mini-FC). Figure S4. Funnel plot for publication bias analysis of specificity of the mini-fluid challenge test (Mini-FC) (DOCX 3448 kb) [file 13054_2019_2545_MOESM1_ESM.docx]
